# Synthesis of Super-High-Viscosity Poly-γ-Glutamic Acid by *pgdS*-Deficient Strain of *Bacillus licheniformis* and Its Application in Microalgae Harvesting

**DOI:** 10.3390/microorganisms12122398

**Published:** 2024-11-22

**Authors:** Xiaohui Zhang, Wei Wu, Hongxiao Mou, Jun Liu, Lei Lei, Xin Li, Dongbo Cai, Yangyang Zhan, Xin Ma, Shouwen Chen

**Affiliations:** 1 School of Life Science and Technology, Wuhan Polytechnic University, Wuhan 430023, China; zhangxiaohui2524@163.com (X.Z.); 1741216705go@gmail.com (W.W.); mouhongxiao1025@163.com (H.M.); junliu85@163.com (J.L.); lei_bc@whpu.edu.cn (L.L.); 2 State Key Laboratory of Biocatalysis and Enzyme Engineering, Environmental Microbial Technology Center of Hubei Province, College of Life Sciences, Hubei University, Wuhan 430062, China; caidongbo@hubu.edu.cn (D.C.); yangyangzhan@hubu.edu.cn (Y.Z.); maxin@hubu.edu.cn (X.M.)

**Keywords:** poly-γ-glutamic acid, *pgdS* gene, super high viscosity, *Bacillus licheniformis*, microalgae flocculation

## Abstract

Poly-γ-glutamic acid (γ-PGA) is a natural polymer whose molecular weight and viscosity are critical for its application in various fields. However, research on super-high-molecular-weight or -viscosity γ-PGA is limited. In this study, the *pgdS* gene in *Bacillus licheniformis* WX-02 was knocked out using homologous recombination, and the batch fermentation performances of the recombinant strain WX-ΔpgdS were compared to those of WX-02. Nitrate accumulation was observed in the early fermentation stages of WX-ΔpgdS, and gene transcription analysis and cell morphology observations revealed that nitrite accumulation was caused by oxygen limitation due to cell aggregation. When the aeration and agitation rates were increased to 2.5 vvm and 600 r/min, respectively, and citrate was used as a precursor, nitrite accumulation was alleviated in WX-ΔpgdS fermentation broth, while γ-PGA yield and broth viscosity reached 17.3 g/L and 4988 mPa·s. Scanning electron microscopy (SEM) showed that the γ-PGA produced by WX-ΔpgdS exhibited a three-dimensional porous network structure. At a γ-PGA concentration of 5 mg/L, the fermentation broth of WX-ΔpgdS achieved a flocculation efficiency of 95.7% after 30 min of microalgae settling. These findings demonstrate that *pgdS* knockout results in super-high-viscosity γ-PGA, positioning it as an eco-friendly and cost-effective biocoagulant for microalgae harvesting.

## 1. Introduction

Poly-γ-glutamic acid (γ-PGA) is a valuable natural biopolymer composed of D- and L-glutamic acid units linked by amide bonds between the γ-carboxyl groups, a structure that significantly differentiates it from other proteins [1]. γ-PGA exhibits unique properties, including biodegradability, water solubility, edibility, and nontoxicity, which make it highly suitable for applications in agriculture, food, pharmaceuticals, cosmetics, and environmental management [2,3,4]. Currently, *Bacillus* species are widely employed for the safe and natural microbial synthesis of γ-PGA. Key characteristics such as molecular weight (Mw), stereochemical composition, and structure primarily determine the biological properties and industrial applications of γ-PGA. For example, γ-PGA with an Mw greater than 1.0 × 10^6^ Da possesses high viscosity and can be developed as a thickener or bioflocculant [5,6], whereas γ-PGA with an Mw lower than 4.0 × 10^5^ Da is preferred for drug delivery applications [7,8]. Therefore, the control of γ-PGA’s Mw and the modification of its stereochemical composition and structure are of fundamental and practical importance for the commercial development of this biopolymer.

Microalgae are an attractive source of feedstock for biofuel production, as well as other bio-products such as omega-3 fatty acids and aquaculture feed. Many techniques are currently applied in microalgae harvesting and recovery, including filtration, flotation, centrifugation, flocculation, and magnetic separation [9]. Among these techniques, flocculation is considered an effective harvesting strategy due to its low energy consumption and total cost [10]. High Mw γ-PGA has been identified as a potential bioflocculant of microalgae [11]. The reported Mw of ultra-high-molecular-weight γ-PGA exceeded 2.0 × 10^6^ Da [6,12], which was still lower than that of commercial polyacrylamide (1.0–2.0 × 10^7^ Da). Researchers have attempted to enhance the flocculation efficiency of γ-PGA by altering the environmental pH [13], grafting it with other polymers such as chitosan [14], or inducing self-crosslinking through γ-radiation or chemical reagents [15,16]. The aforementioned approaches require the addition of external agents, such as pH adjusters or modifiers, which not only increase production costs, but also pose a risk of secondary pollution. In contrast, increasing the Mw or viscosity of γ-PGA during the microbial synthesis stage, rather than post-synthesis modification, would avoid additional production costs, making it a more economically viable option for microalgae harvesting.

Previously, efforts were made to regulate the Mw of γ-PGA during microbial cultivation by optimizing fermentation conditions [17,18]. However, this method is insufficiently precise for controlling the Mw of γ-PGA. To date, regulating the expression of γ-PGA depolymerase has been demonstrated as an efficient way to achieve desirable Mws of γ-PGA [19]. A depolymerase, PgdS (encoded by *pgdS* gene) has been characterized from both *Bacillus subtilis* and *Bacillus licheniformis*, and identified as an endo-type hydrolase responsible for degradation of γ-PGA [20,21]. Our previous research demonstrated that overexpression of the *pgdS* gene from *B. licheniformis* WX-02 resulted in a high γ-PGA yield of 39.1 g/L with a significant reduction in Mw to 7.8 × 10^4^ Da [22], confirming that the *pgdS* gene encodes an endo-type γ-PGA hydrolase. However, studies by different researchers have reported inconsistent findings regarding the effects of *pgdS* deletion on cell growth and γ-PGA production, and the underlying mechanisms responsible for these effects remain unclear [23]. On the other hand, many *Bacillus* species possess a nitrate reduction pathway that first reduces nitrate to nitrite under oxygen-limiting conditions, accompanied by electron transport and ATP production, and subsequently converts nitrite to ammonium [24]. Therefore, nitrate could serve as both a nitrogen source and an electron acceptor. Studies have reported that the appropriate addition of nitrate can enhance γ-PGA synthesis in *B. licheniformis* [25]. Conversely, nitrite, a reduction product of nitrate, has been demonstrated to exhibit toxic effects when accumulated in the fermentation broth, leading to a significant reduction in γ-PGA yield [26,27]. But the effects of *pgdS* deficiency on nitrate and nitrite reduction pathway have not yet been fully understood.

In this study, the *pgdS* gene was knocked out to investigate its significance in vivo, and the fermentation profiles of the recombinant strain WX-ΔpgdS were compared with those of the wild-type strain WX-02. Cell morphology observations and transcriptional analyses were conducted to investigate the potential mechanisms underlying the effects of *pgdS* deletion on γ-PGA fermentation. Furthermore, the characteristics of γ-PGA synthesized by the recombinant strain were analyzed, including Mw, stereochemical composition, and microstructure. Finally, the flocculation efficiencies of microalgae were evaluated using the fermentation broth of WX-ΔpgdS and its purified γ-PGA, and the results were compared with those obtained from the wild-type strain and commercial anionic polyacrylamide. This research provides insights into the intracellular function of the *pgdS* gene and its impact on the synthesis of γ-PGA and other related pathways, while promoting the application of γ-PGA produced by WX-ΔpgdS as an eco-friendly flocculant or coagulant for microalgae harvesting.

## 2. Materials and Methods

### 2.1. Bacterial Strains and Plasmids

The bacterial strains and plasmids used in this study are listed in Table 1. The primers used for strain construction and RT-qPCR are provided in Table S1. *B. licheniformis* WX-02 (CCTCC M208065), a wild-type strain with high γ-PGA production, was employed as the parental strain for constructing the recombinant strain. The plasmid T2(2)-Ori, a temperature-sensitive vector, was applied for gene knockout in *B. licheniformis*. *Escherichia coli* DH5α served as the host for plasmid construction.

### 2.2. Culture and Medium

*B. licheniformis* and *E. coli* were both cultured in Luria–Bertani (LB) medium, and 250 mL flasks containing 50 mL LB liquid medium were maintained at 37 °C in a rotary shaker with 220 r/min for 10 h. When necessary, 20 μg/mL kanamycin was added to the media. The γ-PGA production medium in a 5 L fermenter consisted of (per liter) 80 g of glucose, 40 g of γ-PGA synthetic precursor (L-glutamate, or L-glutamine, or citrate), 15 g of NaNO_3_, 7 g of NH_4_Cl, 0.5 g of MgSO_4_·7H_2_O, 0.5 g of K_2_HPO_4_·3H_2_O, 0.15 g of CaCl_2_, 0.04 g of FeCl_3_, and 0.04 g of MnSO_4_·H_2_O, with a pH of 7.2 [26].

*C.* sp. CMBB266 strains were cultured in modified Endo medium consisting of (per liter) 30 g of glucose, 3 g of KNO_3_, 1.2 g of KH_2_PO_4_, 1.2 g of MgSO_4_·7H_2_O, 0.2 g of trisodium citrate, 0.016 g of FeSO_4_·7H_2_O, 2.1 mg of EDTA-Na_2_, 0.105 g of CaCl_2_·2H_2_O, 2.86 mg of H_3_BO_3_, 0.22 mg of ZnSO_4_·7H_2_O, 1.81 mg of MnCl_2_·4H_2_O, 0.021 mg of Na_2_MoO_4_·2H_2_O, and 0.07 mg of CuSO_4_·5H_2_O, with a pH of 6.5 [28]. *Chlorella* strains were first cultured in 100 mL flasks containing 20 mL of sterile modified Endo medium maintained in the darkness at 30 °C in a rotary shaker with 180 r/min for 5–6 days. Then, the algal cells were inoculated into 500 mL flasks containing 200 mL modified Endo medium with an inoculum volume of 1% (*v*/*v*), and grown with the same culture conditions above for 6 days.

### 2.3. Knockout of pgdS Gene in B. licheniformis

The method for gene deletion in *B. licheniformis* WX-02 followed our previous study [29], and the procedure is briefly described as follows. First, the upstream and downstream homogenous arms of the *pgdS* gene were amplified from the genomic DNA of *B. licheniformis* WX-02 using primers *pgdS*-AF/AR and *pgdS*-BF/BR and fused using splicing overlap extension (SOE)-PCR. The fused fragment was inserted into the plasmid T2(2)-Ori at the restriction enzyme sites *Xba*I/*Sac*I. Diagnostic PCR and DNA sequencing were used to confirm the successful construction of the plasmid T2-PgdS.

Then, the plasmid T2-PgdS was electro-transferred into *B. licheniformis* WX-02, and verified by diagnostic PCR and plasmids extraction. T2(2)-Ori is a temperature-sensitive plasmid, with intracellular plasmid replication inhibited at high temperatures of 45 °C. Only strains that undergo single-crossover recombination retain kanamycin resistance under these conditions. Transformants containing the free plasmid T2-PgdS were first cultivated in LB liquid medium with 20 μg/mL kanamycin at 45 °C. Subsequently, the single-crossover mutants were transferred to kanamycin-free medium and incubated at 37 °C for several generations to facilitate double-crossover recombination. After double-crossover recombination, only the homologous arm sequences remained at the target gene site, while the kanamycin resistance gene was not integrated into the genome. As a result, double-crossover strains became sensitive to kanamycin. The kanamycin-sensitive colonies were selected and further verified by diagnostic PCR to confirm successful double-crossover events. The recombinant strain was confirmed by DNA sequencing and designated WX-ΔpgdS.

### 2.4. Batch Cultures in 5 L Stirred Fermenters

The γ-PGA fermentations were conducted in 5 L mechanically stirred fermenters (T&J Bio-engineering, Shanghai, China) referring to our previous study [27]. The seed culture was inoculated at a 3% (*v*/*v*) ratio. The fermenters were equipped with online dissolved oxygen (DO), pH, and temperature electrodes for monitoring relevant parameters. The initial pH was adjusted to 7.2 ± 0.1 using a 6 M aqueous ammonia solution. During fermentation, the pH was maintained between 6.5 and 7.5 by adding aqueous ammonia or hydrochloric acid (concentration of 6 M). The fermentation medium volume was 2.5 L, and the culture temperature was held at 37 °C. The agitation speed and aeration rate were initially set at 400 r/min and 1.0 vvm and remained constant under the normal oxygen supply conditions. When the high oxygen supply conditions were implemented, the agitation speed and aeration rate were increased to 600 r/min and 2.5 vvm, respectively, at 12 h of fermentation.

### 2.5. Analytic Methods

Biomass was quantified by measuring the optical density at 600 nm, while glucose and L-glutamate concentrations in the broth were determined using an SBA-40C bioanalyzer (Academy of Sciences, Jinan, Shandong, China) [27]. Nitrate and nitrite concentrations were determined by the salicylic acid method and diazo coupling procedure, respectively [30,31]. The viscosity of the fermentation broth and 1% (*w*/*w*) γ-PGA solutions were measured using a rotational viscometer. The concentration of γ-PGA in the broth was determined by the cetylmethylammonium bromide (CTAB) assay [32].

For γ-PGA purification, the fermentation broth was mixed with two volumes of distilled water, adjusted to pH 2.5, and centrifuged at 12,000 r/min for 10 min. Then, the broth pH was readjusted to 7.0, and three volumes of absolute ethanol were added. The resulting precipitate was freeze-dried under vacuum at −50 °C. The weight-average molecular weights of the purified γ-PGA samples were measured using gel permeation chromatography (GPC) with a refractive index (RI) detector and a Shodex (Tokyo, Japan) OHpak SB-806 HQ column (8.0 mm ID × 300 mm, 13 mm) [22]. To analyze the stereochemical composition, γ-PGA underwent acid hydrolysis and amino acid derivatization were carried out according to the previously reported methods [33]. The concentrations of D/L-glutamate in the hydrolyzed samples were determined by high-performance liquid chromatography (HPLC) using an Agilent (Santa Clara, CA, USA) ZORBAX Eclipse Plus C18 column (4.6 mm × 250 mm, 5 μm) at 340 nm. Mobile phase A consisted of 50 mmol/L KH_2_PO_4_ (pH 2.7), acetonitrile, and methanol in a volume ratio of 18:1:1, at a flow rate of 0.5 mL/min, while mobile phase B contained the same components in a volume ratio of 12:7:1, at the same flow rate.

All samples were analyzed in triplicate, with the results reported as the mean ± the standard deviation for each data point.

### 2.6. Observation of Cell and γ-PGA Morphology

After 12 h of fermentation, 10 mL of broth was sampled from the fermenter and centrifuged at 10,000 r/min for 5 min to collect bacterial cells. Normal saline was used to remove surface mucus from the cells. The bacterial suspension was flame-fixed onto a glass slide and stained using the Gram staining method. Bacterial cell morphology was observed using an inverted microscope (Leica DW2500, Wetzlar, Germany).

The surface morphology of γ-PGA samples was observed using a scanning electron microscope (SEM, Quattro S, Thermo Fisher Scientific, Hamburg, Germany) [34]. Purified and lyophilized γ-PGA samples were mounted on stubs, sputter-coated with gold, and observed at various magnifications. The γ-PGA samples produced by WX-02 and WX-ΔpgdS were analyzed at three different points to ensure consistency in the observed morphologies.

### 2.7. Transcriptional Analysis

Cells from the wild-type and recombinant strains were sampled after 12 h of fermentation. RT-qPCR was performed according to the previous research [27]. Briefly, total RNA was extracted using TRIzol^®^ Reagent (Invitrogen, Waltham, MA, USA), and trace DNA was digested with RNase-free DNase I enzyme (TaKaRa, Kyoto, Japan). The first strand of cDNA was synthesized using the RevertAid First Strand cDNA Synthesis Kit (Thermo, Waltham, MA, USA). Real-time PCR was conducted using SYBR^®^ Select Master Mix (Thermo, Waltham, MA, USA) according to the manufacturer’s instructions. The transcriptional levels of genes in the recombinant strain (WX-ΔpgdS) were compared with those of the control strain (WX-02) after normalizing to the reference gene 16S rRNA. All the samples were measured in triplicates, and the data were presented as the mean ± the standard deviation.

### 2.8. Flocculation of Microalgae Culture

The flocculation of microalgae cultures was conducted following the established protocols [13]. Generally, flocculation experiments were carried out with culture volumes of 100 mL distributed in 150 mL cups. The pH of the samples was maintained between 6.0 and 7.0 without adjustment. A concentration of 50 mg/L of polysilicate aluminum ferrite (PSAF) and a specific amount of organic polymer coagulant were sequentially added to the microalgae culture, followed by flash mixing at 300 r/min for 1 min, and then slow mixing at 100 r/min for 2 min. The microalgal suspensions were left to settle at room temperature without agitation. Subsequently, the supernatants were pipetted from half the height of the clarified layer, and used to measure the optical density at 685 nm (OD685 nm). Flocculation efficiency (FE) for each test was calculated according to the following equation:

$$FE\left(\%\right)=\frac{A_{0}-A_{1}}{A_{0}}\times 100\%$$

where A_0_ and A_1_ represent the optical density values at 685 nm of the microalgal suspension before and after flocculation, respectively.

## 3. Results

### 3.1. Disruption of pgdS and Its Effect on γ-PGA Production

**Figure 1:**
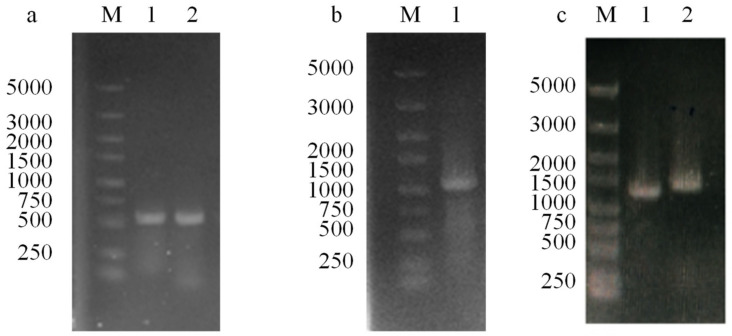
PCR amplification and colony PCR verification of knockout elements. (**a**) PCR products of the homologous arms and the Splicing fragment; Lane M: DL5000 DNA Marker; Lane 1: PCR product of the upstream homologous arm of *pgdS* (538 bp); Lane 2: PCR product of the downstream homologous arm of *pgdS* (535 bp). (**b**) SOE-PCR product of the up and the down homologous arm of *pgdS*; Lane 1: SOE-PCR product (1073 bp). (**c**) Confirmation of the DH5α/T2-pgdS and the *pgdS*-deficient strain; Lane 1: PCR product of DH5α/T2-pgdS (1400 bp); Lane 2: PCR product of WX-02-ΔpgdS by primers *pgdS*-YF/*pgdS*-YR (1493 bp).

In this study, the *pgdS* gene in *B. licheniformis* WX-02 was disrupted using the homologous recombination method. Figure 1 presents the PCR validation of the upstream and downstream homologous arms, the recombinant plasmid T2-pgdS, and the *pgdS* gene knockout. As a result, the recombinant strain *B. licheniformis* WX-ΔpgdS was successfully constructed.

**Figure 2:**
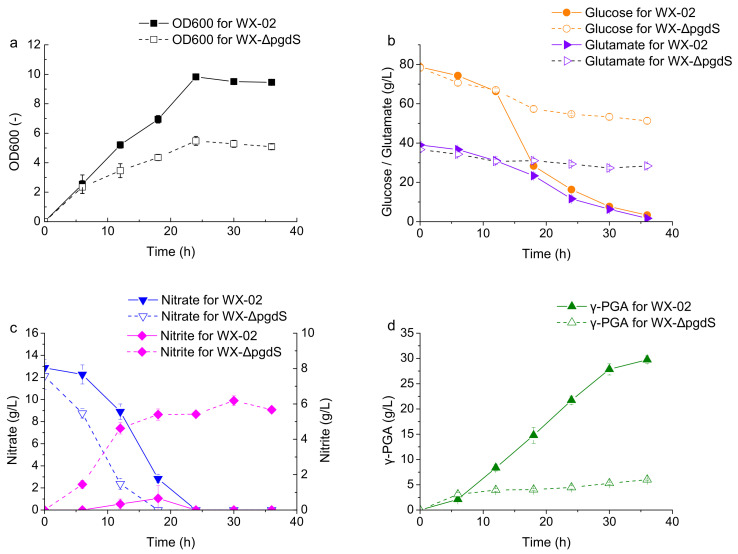
Profile comparison of batch fermentations between WX-02 and WX-ΔpgdS. (**a**) OD600; (**b**) concentrations of glucose and glutamate; (**c**) concentrations of nitrate and nitrite; (**d**) γ-PGA yields.

The fermentation profiles of the wild-type strain WX-02 and the recombinant strain WX-ΔpgdS were subsequently compared to investigate the effect of *pgdS* disruption on cell metabolism, particularly γ-PGA synthesis. As shown in Figure 2, the cell growth rate of WX-ΔpgdS was significantly slower than that of WX-02 after 6 h, and the A600 value of WX-ΔpgdS was 5.08 at the end of fermentation, representing a 46.2% decrease compared to that of WX-02. Additionally, the consumption of glucose and glutamate by the recombinant WX-ΔpgdS nearly ceased after 12 h of fermentation, with a concomitant significant reduction in the rate of γ-PGA synthesis. By the end of fermentation, 65.0% of glucose and 77.1% of glutamate remained in the fermentation broth of WX-ΔpgdS, and the γ-PGA yield was only 6.0 g/L. In contrast, these substrates were almost completely consumed in the fermentation broth of WX-02, where the final γ-PGA production reached 29.7 g/L, nearly five-fold higher than that of WX-ΔpgdS.

By contrast, nitrate was consumed more quickly by WX-ΔpgdS compared to WX-02 during the early stages of fermentation. Simultaneously, nitrite concentration accumulated rapidly in the fermentation broth of WX-ΔpgdS, reaching a sustained high level of 5.0 g/L until the end of fermentation. This phenomenon of nitrite accumulation has been previously observed in glycerol-based fermentation [27]. Thus, it is reasonable to hypothesize that nitrite accumulation following *pgdS* deletion may be a key factor in inhibiting substrate assimilation and γ-PGA production.

### 3.2. Effects of pgdS Deficiency on Cell Morphology and the Transcription of Genes Related to Respiration

To investigate the mechanism of excessive nitrite accumulation caused by *pgdS* deficiency, cell morphology was observed alongside the transcription levels of key genes involved in aerobic and nitrate respiration. NADH dehydrogenase (encoded by *ndh*), cytochrome bd ubiquinol oxidase (encoded by *cydABCD*), and cytochrome aa3 oxidase (encoded by *qoxABCD*) are key enzymes involved in aerobic respiration [35]. The *narGHJI* gene cluster encodes respiratory nitrate reductase, while *nasBC* and *nasDE* encode assimilatory nitrate reductase and nitrite reductase, respectively [36]. Fnr, recognized as an anaerobic transcription factor, is activated under anaerobic or microaerobic conditions [37].

**Figure 3:**
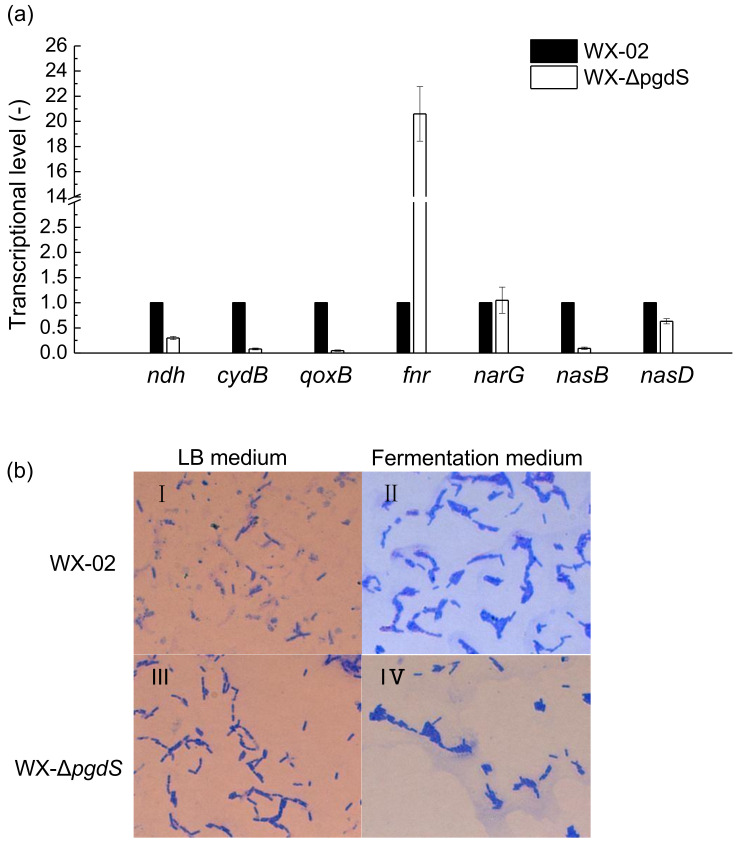
Transcriptional levels of genes involved in aerobic and nitrate respiration, and observation of cell morphology. (**a**) Transcriptional levels; (**b**) cell morphology.

The transcriptional levels of *ndh*, *cydB*, and *qoxB* decreased by 0.3-fold, 0.08-fold, and 0.05-fold, respectively, in the recombinant strain compared to the wild-type strain, as shown in Figure 3a. In contrast, the transcriptional levels of *narG* and *nasD* in WX-ΔpgdS were almost identical to those in WX-02. Furthermore, *fnr* transcription in WX-ΔpgdS increased significantly by 20.6-fold compared to that in WX-02, indicating that the recombinant strains were under severe anaerobic conditions. However, the γ-PGA yield and the viscosity of the fermentation broth produced by the recombinant strain were significantly lower than those of the wild-type strain, and further research is required to fully explain these findings.

On the other hand, the wild-type strains formed aggregates at 12 h when the concentration of γ-PGA in the broth was 8.4 g/L, as shown in Figure 3b. Comparatively, the recombinant strain also exhibited aggregation, even though the γ-PGA concentration produced by the recombinant was only 4.0 g/L at 12 h. In LB medium, the wild-type strain exhibited uniform dispersion, whereas the recombinant strain remained aggregated. It is well known that cell aggregation adversely affects the transfer of substrates and oxygen to cells within the aggregates, resulting in inefficient substrates and oxygen absorption [38]. Thus, the aggregation of recombinant cells likely limited oxygen transfer, leading to reduced transcription of aerobic respiration genes and increased expression of the anaerobic transcription factor *fnr*, ultimately causing nitrite accumulation.

### 3.3. γ-PGA Enhancement of WX-ΔpgdS by Optimization of Precursor Substrate and Oxygen Supply Conditions

In this study, three different precursor substrates including L-glutamate, L-glutamine, and sodium citrate were used in batch fermentations with both WX-ΔpgdS and WX-02. Additionally, high oxygen supply conditions were implemented by increasing the agitation speed and aeration rate, aiming to alleviate oxygen insufficiency during the early fermentation state in the mutant strain, and address the issue of nitrite accumulation.

When glutamine or citrate was used as the main precursor substrate instead of glutamate, the nitrate consumption rate was relatively slower, and nitrite accumulated more gradually in the early stage of fermentation. However, nitrite still accumulated to high levels (>3.0 g/L) under regular agitation speeds and aeration rates, with γ-PGA production reaching 3.7 and 5.5 g/L in the glutamine-based and the citrate-based medium, respectively, which had no significant improvement over the glutamate-based medium (Figure S1).

**Figure 4:**
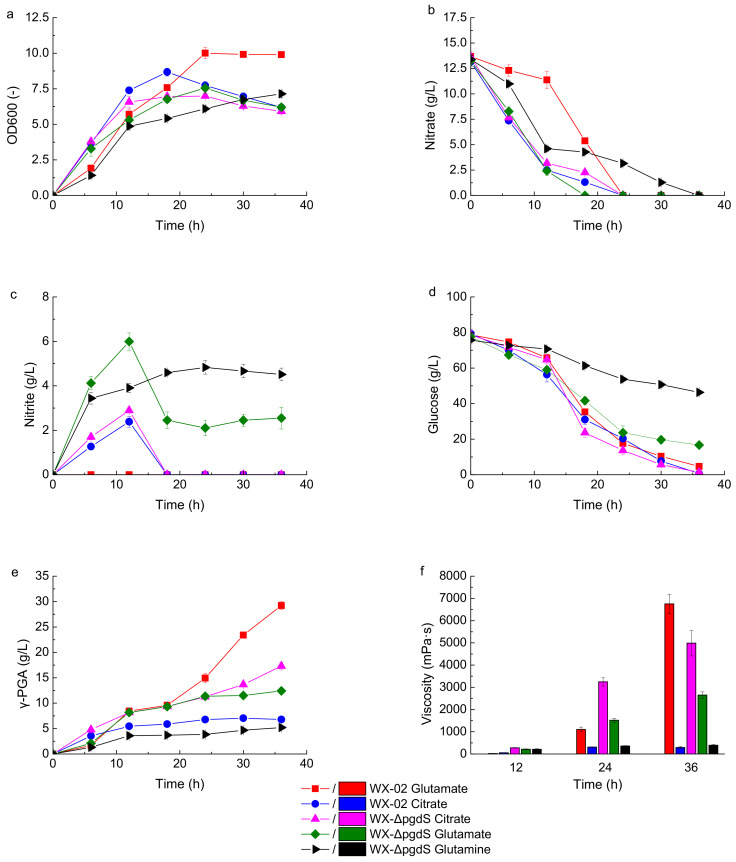
The fermentation profiles of WX-ΔpgdS and WX-02 using different precursor substrates under high oxygen supply conditions. (**a**) OD600; (**b**) nitrate; (**c**) nitrite; (**d**) glucose; (**e**) γ-PGA yield; (**f**) broth viscosity.

After increasing the agitation speed and aeration rate to 600 r/min and 2.0 vvm at 12 h, the nitrite concentration in the glutamate-based medium sharply declined from 6.0 g/L at 12 h to 2.5 g/L at 18 h, stabilizing between 2.0 and 2.5 g/L (Figure 4c). Under high oxygen supply conditions, glucose consumption by WX-ΔpgdS reached 61.0 g/L, a 126% increase compared to regular conditions, although 16.7 g/L of unused glucose still remained in the fermentation broth. The final γ-PGA yield in the glutamate-based medium by WX-ΔpgdS arrived 12.4 g/L, a notable increase of 106.5% compared to pre-optimization levels. Furthermore, nitrite absorption was more effective when glutamine or citrate was used instead of glutamate. Notably, nitrite in the citrate-based medium became undetectable after 18 h, with a residual glucose concentration of only 1.3 g/L at the end of fermentation. The γ-PGA yield in the citrate-based medium reached 17.3 g/L, the highest among the three tested media for the recombinant strain (Figure 4e).

These results indicated that increasing the aeration rate and stirring speed, combined with using citrate instead of glutamate as a precursor, could effectively mitigate nitrite accumulation, enhance glucose consumption, and improve γ-PGA production by WX-ΔpgdS. Additionally, the final viscosity of WX-ΔpgdS cultured in the citrate-based medium reached 4988 mPa·s, comparable to that of WX-02 cultured in the glutamate-based medium (Figure 4f). However, the γ-PGA yield by WX-ΔpgdS was only 59.2% of that produced by WX-02, suggesting that the characteristics of the γ-PGA produced by the recombinant strain may have been altered.

### 3.4. Effects of pgdS Deficiency on the Characteristics of γ-PGA

After purification, γ-PGAs produced by WX-02 and WX-ΔpgdS were used to prepare 1% γ-PGA solutions for rotational viscosity measurement, as shown in Figure S2. The viscosity of the γ-PGA solution from WX-ΔpgdS reached 865 mPa·s, which was 3.1-fold higher than that of the solution from WX-02. An increase in the viscosity of a biopolymer is generally associated with either an increase in its Mw or a change in its topological structure. Therefore, gel permeation chromatography (GPC), the most common method for Mw determination, was conducted. Unexpectedly, the elution times of γ-PGAs produced by WX-ΔpgdS and WX-02 were 11.013 and 11.059 min, respectively, indicating that the average Mws of the γ-PGA produced by the wild-type and mutant strains were nearly identical.

Next, the stereochemical compositions of γ-PGAs from both strains were analyzed, and results are showed in Figure S2. Calculations showed that γ-PGA produced by WX-02 contained 84.8% D-glutamic acid and 15.2% L-glutamic acid, while γ-PGA from WX-ΔpgdS contained 86.2% D-glutamic acid and 13.8% L-glutamic acid, indicating that D- and L-glutamic acid contents of both γ-PGAs were not significantly different. These results demonstrate that the high viscosity of the γ-PGA produced by WX-ΔpgdS was neither due to an increase in Mw, nor changes in stereochemical composition.

**Figure 5:**
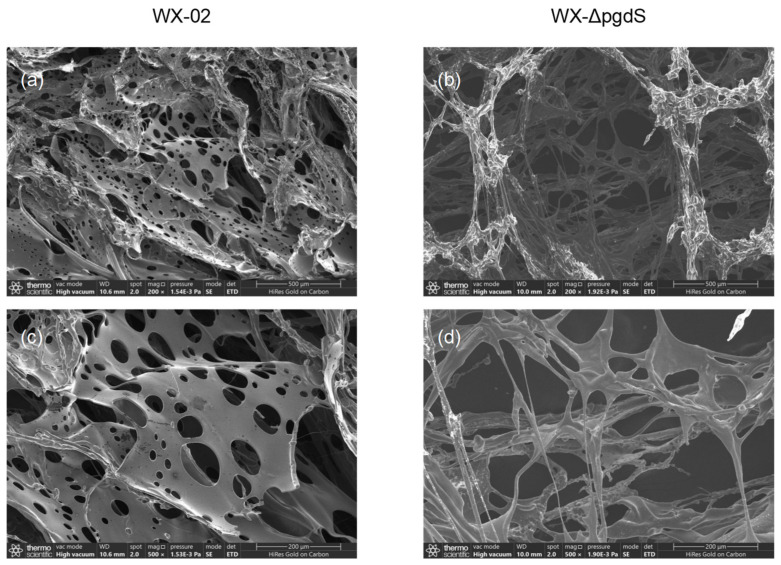
SEM images of surface of γ-PGA produced by WX-02 and WX-ΔpgdS, respectively. (**a**) Image of γ-PGA produced by WX-02 with scale bar of 500 μm; (**b**) image of γ-PGA produced by WX-ΔpgdS with scale bar of 500 μm; (**c**) image of γ-PGA produced by WX-02 with scale bar of 200 μm; (**d**) image of γ-PGA produced by WX-ΔpgdS with scale bar of 200 μm.

In addition to Mw and stereochemical composition, the microstructure of a bioplymer may also influence its viscosity. Therefore, SEM was employed to observe the surface morphology and microstructure of the γ-PGA, as shown in Figure 5. The γ-PGA produced by WX-02 exhibited multiple smooth layers with few vertical cross-links between layers. Numerous pores were present on each layer, with most pore sizes smaller than 100 μm. In contrast, the γ-PGA produced by WX-ΔpgdS formed an interconnected three-dimensional porous network structure, with significantly larger pores than those observed on the smooth layers of WX-02. This three-dimensional porous network structure has been observed in hydrophilic gel made from modified γ-PGA, which exhibit superior water retention properties [3,39].

These findings suggest that the γ-PGA endohydrolase encoded by the *pgdS* gene disrupts the three-dimensional porous network structure of γ-PGA, thereby reducing its viscosity. When the *pgdS* gene was knocked out, a super-high-viscosity γ-PGA was generated, which led to cell aggregation, resulting in oxygen insufficiency and nitrite accumulation.

### 3.5. Harvesting Microalgae Using Fermentation Broth Containing Super-High-Viscosity γ-PGA

**Figure 6:**
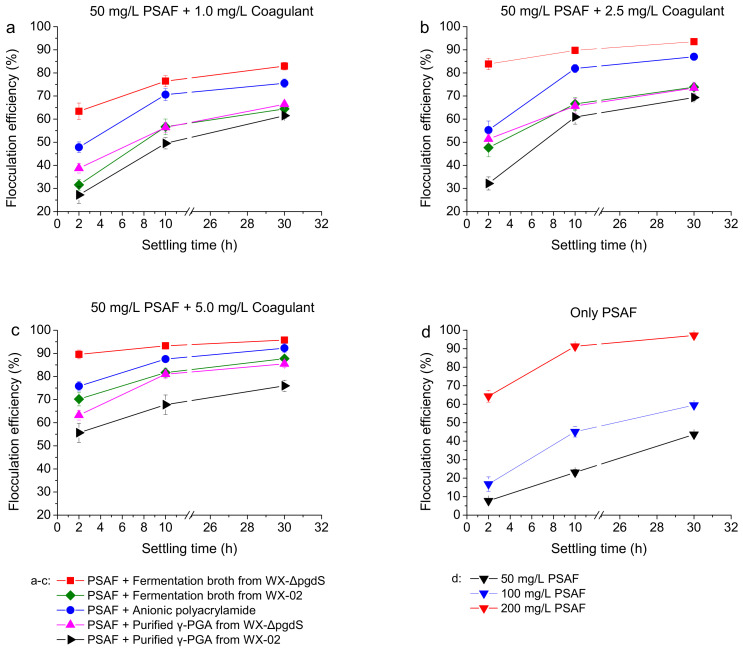
Flocculation efficiency comparison between fermentation broths, purified γ-PGAs and commercial anionic polyacrylamide. (**a**) Adding 50 mg/L PSAF and 1.0 mg/L coagulant; (**b**) adding 50 mg/L PSAF and 2.5 mg/L coagulant; (**c**) adding 50 mg/L PSAF and 5.0 mg/L coagulant; (**d**) only adding PSAF (50, 100, 200 mg/L).

In this study, a concentration of 50 mg/L polysilicate aluminum ferrite was used to flocculate *Chlorella vulgaris*, in combination with biocoagulants such as fermentation broths or purified γ-PGAs derived from different strains. Commercial anionic polyacrylamide was used as a control for comparison with the γ-PGA coagulants. As shown in Figure 6, when purified γ-PGA was added in the microalgae suspension at concentrations of 2.5 and 5.0 mg/L, the flocculation efficiency of the γ-PGA produced by WX-ΔpgdS reached 51.4% and 63.2%, respectively, after 2 min settling, representing increases of 59.6% and 13.7%, compared to the wild-type strain.

In comparison, when the fermentation broth of WX-ΔpgdS was added to the microalgal suspension at a γ-PGA concentration of 2.5 mg/L, the flocculation efficiency reached 83.8% after 2 min, 1.76-fold and 1.52-fold higher than that of WX-02 fermentation broth and anionic polyacrylamide, respectively. As shown in Figure S3, the microalgal suspension treated with WX-ΔpgdS fermentation broth was significantly clearer than those treated with other coagulants after 2 min settling. When the settling time reached 30 min, a flocculation efficiency of 93.5% was achieved using the recombinant fermentation broth. Furthermore, the flocculation efficiency of WX-ΔpgdS fermentation broth reached 89.5% at 2 min and 95.7% at 30 min when the γ-PGA concentration was increased to 5.0 mg/L. The flocculation efficiency of WX-ΔpgdS fermentation broth was consistently the highest among all experimental groups under the same conditions.

These results indicate that the γ-PGA produced by the *pgdS*-deficient mutant exhibits higher flocculation efficiency than that produced by the wild-type strain, and the utilization of high-viscosity fermentation broth results in better flocculation efficiency than purified γ-PGA. The flocculation efficiency of fermentation broth has been reported to be higher than that of the commercial purified γ-PGA, as the broth contains not only γ-PGA but also other metabolites like polysaccharides that may enhance the flocculation process [13]. Using fermentation broth as a bioflocculant or biocoagulant is more cost-effective, is nontoxic in nature, and avoids the problem of secondary pollution.

## 4. Discussion

Since γ-PGAs with varying Mws and viscosities can be utilized for diverse applications, many researchers aim to precisely regulate the related characteristics of γ-PGA. Based on this goal, several potential γ-PGA hydrolases have been characterized from various *Bacillus* strains, including PgdS [20,40,41], CwlO [42], Ggt [43], and some others [19]. Among these hydrolases, PgdS has been identified as an endo-type γ-PGA hydrolase that efficiently cleaves the γ-glutamyl bond between D- and L-glutamic acid residues of γ-PGA. Studies have shown that Mw reductions of γ-PGA, from over 10^6^ Da to 10^4^ Da or even lower, could be achieved by overexpressing the *pgdS* gene [21,44], optimizing the expression elements (promoters and signal peptides) of the *pgdS* gene [22], and co-expressing other genes related to γ-PGA synthesis [45], which also enhanced γ-PGA yields. However, reported results on the effects of *pgdS* gene deficiency on cell growth and γ-PGA synthesis have been conflicting. Scoffone et al. reported that knocking out the *pgdS* gene had no significant impact on cell growth, γ-PGA yield, or Mw in *B. subtilis* [46]. By contrary, Ojima et al. found that knocking out the *pgdS* gene limited γ-PGA synthesis, but the mechanism of this inhibitory effect was not elucidated [23]. In this study, a significant reduction in γ-PGA yield was also observed in batch fermentation of the *pgdS*-deficient strain compared to the wild-type strain. Therefore, we aimed to explore both extrinsic and intrinsic factors contributing to the inhibition of cell growth and γ-PGA production due to *pgdS* gene deletion.

In our earlier research, nitrite accumulation was observed when glycerol was used as a carbon source instead of glucose, leading to cell growth inhibition and reduced γ-PGA yield [27]. Coincidentally, Ojima et al. also employed nitrate in the fermentation medium, as in this study. Nitrite measurements confirmed that high concentrations of accumulated nitrite were likely the primary extrinsic factor causing the inhibitory effects in the fermentation process of WX-ΔpgdS. Additionally, the *pgdS*-deficient strains exhibited increased cell aggregation, not only in fermentation broth but also in conventional LB medium. Previous studies reported that γ-D-PGA was tightly anchored to the cell wall in *B. anthracis* in the absence of the *capD* gene, which also encodes a γ-PGA endo-hydrolase similar to *pgdS* [47,48]. This led us to hypothesize that unreleased γ-PGA anchored to the cell wall facilitated cell aggregation, negatively affecting oxygen transfer to aggregated cells. Gene transcription analysis confirmed this hypothesis, showing that the transcription levels of respiration-related genes were significantly downregulated, while the global anaerobic regulator Fnr was markedly upregulated. Cai et al. also observed nitrite accumulation in the culture broth of an Fnr overexpression strain [26]. It is suggested that early entry into anaerobic nitrate respiration significantly increases the likelihood of nitrite accumulation. Based on these findings, we increased the initial agitation speed and aeration rate, combined with using critic acid as the precursor substrate, which significantly alleviated nitrite accumulation and improved γ-PGA production to 17.3 g/L. Although this yield is lower than that of the wild-type strain, it remains the highest reported for a *pgdS*-deficient strain.

The biodegradable and nontoxic nature of γ-PGA makes it an eco-friendly option for wastewater treatment, particularly in downstream food fermentation processes or microalgae harvesting [4]. It has been demonstrated that polymers with longer chain lengths or more branched structures possess greater surface area and more adsorption sites, enhancing flocculation sedimentation rates and leading to denser flocs [49]. However, compared to traditional chemical organic polymer flocculants such as polyacrylamide, γ-PGA has certain disadvantages, for example, relatively lower Mw and fewer branches. Some researchers have attempted to increase the chain lengths or branch numbers of γ-PGA, such as by adding agents to form cross-linked γ-PGA [15,16], or grafting with other biopolymers [14], which have indeed improved the flocculation activity of γ-PGA. However, these methods increase the complexity of γ-PGA-flocculant fabrication and significantly raise the cost of raw materials. In this study, SEM images revealed that the γ-PGA produced by WX-ΔpgdS exhibited a three-dimensional porous network structure. Its solution had much higher viscosity than that of WX-02. After purification, this γ-PGA exhibited higher flocculation efficiency than that of the wild-type strain. Moreover, the WX-ΔpgdS fermentation broth was found to be an even better option for flocculating microalgae, achieving flocculation efficiencies of 93.5% or 95.7% at 30 min settling with only 2.5 or 5.0 mg/L of γ-PGA. Considering multiple factors affecting flocculation costs, including the working concentration of γ-PGA, the initial turbidity and pH range of the suspension, the settling time, and the flocculation efficiency, the results of this study are highly competitive compared to similar reports, as listed in Table 2. Based on prior cost estimates reported in the literature [27], the production cost of γ-PGA by WX-ΔpgdS in the fermentation broth was estimated to be approximately USD 5000 per ton, which increased to around USD 12,000 per ton after crude extraction. Both values are significantly higher than the price of commercial anionic polyacrylamide (about USD 1500 per ton). However, it is well known that the monomer of polyacrylamide, acrylamide, is a neurotoxin with substantial risks to human health. In comparison, γ-PGA is harmless to humans, naturally biodegradable, and its degradation products, D- or L-glutamic acid, serve as nutrients for organisms, which eliminates concerns about environmental pollution. Moreover, γ-PGA has been demonstrated to exhibit excellent thermal stability and remain stable in aqueous media under neutral to mildly alkaline conditions [50]. The comparison with commercial chemical polymers clearly highlights the environmental benefits of γ-PGA and its disadvantage in production cost. This underscores the need for further investigation into the synthesis mechanisms of γ-PGA, as well as the development of fermentation technologies to enhance the production of high viscosity γ-PGA. Additionally, due to its safety, environmental friendliness, and cost effectiveness, *Bacillus* fermentation broth likely offers significant practical feasibility for industrial microalgae harvesting.

## 5. Conclusions

This study demonstrated that knocking out the *pgdS* gene generated super-high-viscosity γ-PGA, leading to oxygen insufficiency and nitrite accumulation. Under high oxygen supply conditions, combined with the use of citrate as a precursor instead of glutamate, nitrite accumulation was effectively alleviated during the fermentation of WX-ΔpgdS, and the γ-PGA yield increased to 17.3 g/L, with a high-viscosity broth measuring 4988 mPa·s. The flocculation efficiency of this broth reached 95.7% after 30 min of microalgae settling, positioning it as an eco-friendly and cost-effective option for microalgae harvesting.

## Figures and Tables

**Table 1:** The strains and plasmids used in this research.

Strains and Plasmids	Relevant Characteristics	Source of Reference
Strains		
*E. coli* DH5α	*supE*44 Δ*lac*U169 (f 80 *lacZ*ΔM15) *hsd*R17 *recA*1 *gyrA*96 *thi*1 *relA*1	Lab collection
*B*. *licheniformis* WX-02	Wide-type host strain (CCTCC M208065)	Lab collection
WX-ΔpgdS	Knockout of *pgdS* gene in WX-02	This study
*Chlorella* sp. CMBB266	A freshwater protein-yielding *Chlorella* strain	Provided by Dr. Hu Jin
Plasmids		
T2(2)-Ori	*E. coli*–*B. licheniformis* shuttle vector, kanamycin resistance, for gene knockout	Lab collection
T2-PgdS	T2(2)-Ori derivative containing homologous arms of *pgdS* to block γ-DL-glutamyl hydrolase synthesis	This study

**Table 2:** Comparison of flocculation conditions and efficiencies using different kinds of γ-PGA flocculants.

Flocculant Component and Dosage	Flocculating Object	Initial Turbidity	pH	Settling Time	Flocculation Efficiency	References
PGa21Ca 80–90 mg/L (calcium sulfate, 87%; cross-linked γ-PGA, 5.26%)	Raw water	100 NTU	-	15 min	89.74–96.89%	[51]
Chitosan, 50 mg/L; γ-PGA, 50 mg/L	Potato starch wastewater	-	6.0	15 min	98.30%	[14]
Cross-linked γ-PGA, 2 mg/L; FeCl3, 16 mg/L	*Escherichia* coli	A660 = 0.02	5.0	4 h	88.80%	[16]
*B. licheniformis* broth, 2.5 mL/L	*Desmodesmus* sp. F51	A685 = 1.67	3.0	20 min	99.35%	[13]
Commercial γ-PGA, 22.03 mg/L	*Chlorella vulgaris*	A680 = 1.01	7.5	2 h	91%	[52]
Commercial γ-PGA, 19.08 mg/L	*Chlorella protothecoides*	A680 = 1.08	7.5	2 h	98%	
PSAF, 50 mg/L; *B. licheniformis* WX-ΔpgdS broth, 125 μL/L (γ-PGA = 2.5 mg/L)	*Chlorella vulgaris*	A680 = 1.04	7.0	2 min	83.80%	In this study
				30 min	93.50%	
PSAF, 50 mg/L; *B. licheniformis* WX-ΔpgdS broth, 250 μL/L (γ-PGA = 5.0 mg/L)	*Chlorella vulgaris*	A680 = 1.08	7.0	2 min	89.50%	In this study
				30 min	95.70%	

## Data Availability

The original contributions presented in the study are included in the article/Supplementary Material, further inquiries can be directed to the corresponding authors.

## Supplementary Materials

The following supporting information can be downloaded at: https://www.mdpi.com/article/10.3390/microorganisms12122398/s1, Table S1: Primers used for PCR and qPCR in this study; Figure S1: The fermentation profiles of WX-ΔpgdS using different precursor substrates under regular oxygen supply conditions; Figure S2: Characteristics assay of γ-PGA produced by WX-ΔpgdS and WX-02; Figure S3: Flocculation of microalgae suspension.

## References

[1] Wang L., Chen S., Yu B. (2022). Poly-γ-glutamic acid: Recent achievements, diverse applications and future perspectives. Trends Food Sci. Technol..

[2] Pal P., Singh A.K., Sarangi P.K., Sahoo U.K., Singh H.B., Subudhi S., Singh T.A. (2024). Production of gamma–polyglutamic acid microgel by *Bacillus* species: Industrial applications and future perspectives. Polym. Adv. Technol..

[3] Park S.-B., Sung M.-H., Uyama H., Han D.-K. (2021). Poly (glutamic acid): Production, composites, and medical applications of the next-generation biopolymer. Prog. Polym. Sci..

[4] Elbanna K., Alsulami F.S., Neyaz L.A., Abulreesh H.H. (2024). Poly (γ) glutamic acid: A unique microbial biopolymer with diverse commercial applicability. Front. Microbiol..

[5] Luo Z., Guo Y., Liu J., Qiu H., Zhao M., Zou W., Li S. (2016). Microbial synthesis of poly-γ-glutamic acid: Current progress, challenges, and future perspectives. Biotechnol. Biofuels.

[6] Zhao C., Zhang Y., Wei X., Hu Z., Zhu F., Xu L., Luo M., Liu H. (2013). Production of ultra-high molecular weight poly-γ-glutamic acid with *Bacillus licheniformis* P-104 and characterization of its flocculation properties. Appl. Biochem. Biotechnol..

[7] Liu Z., He Y., Ma X. (2024). Preparation, Characterization and drug delivery research of γ-polyglutamic acid nanoparticles: A review. Curr. Drug Deliv..

[8] Tan J., Wang H., Xu F., Chen Y., Zhang M., Peng H., Sun X., Shen Y., Huang Y. (2017). Poly-γ-glutamic acid-based GGT-targeting and surface camouflage strategy for improving cervical cancer gene therapy. J. Mater. Chem. B.

[9] Fuad N., Omar R., Kamarudin S., Harun R., Idris A., WAKG W.A. (2018). Mass harvesting of marine microalgae using different techniques. Food Bioprod. Process..

[10] Mubarak M., Shaija A., Suchithra T. (2019). Flocculation: An effective way to harvest microalgae for biodiesel production. J. Environ. Chem. Eng..

[11] Najar I., Das S. (2015). Poly-glutamic acid (PGA)-Structure, synthesis, genomic organization and its application: A Review. Int. J. Pharm. Sci. Res..

[12] Zeng W., Liu Y., Shu L., Guo Y., Wang L., Liang Z. (2024). Production of ultra–high–molecular–weight poly–γ–glutamic acid by a newly isolated *Bacillus subtilis* strain and genomic and transcriptomic analyses. Biotechnol. J..

[13] Ndikubwimana T., Zeng X., Liu Y., Chang J.-S., Lu Y. (2014). Harvesting of microalgae *Desmodesmus* sp. F51 by bioflocculation with bacterial bioflocculant. Algal Res..

[14] Li M., Zhu X., Yang H., Xie X., Zhu Y., Xu G., Hu X., Jin Z., Hu Y., Hai Z. (2020). Treatment of potato starch wastewater by dual natural flocculants of chitosan and poly-glutamic acid. J. Clean. Prod..

[15] Taniguchi M., Kato K., Shimauchi A., Xu P., Fujita K.-I., Tanaka T., Tarui Y., Hirasawa E. (2005). Physicochemical properties of cross-linked poly-γ-glutamic acid and its flocculating activity against kaolin suspension. J. Biosci. Bioeng..

[16] Taniguchi M., Kato K., Matsui O., Ping X., Nakayama H., Usuki Y., Ichimura A., Fujita K.-i., Tanaka T., Tarui Y. (2005). Flocculating activity of cross-linked poly-γ-glutamic acid against bentonite and *Escherichia coli* suspension pretreated with FeCl_3_ and its interaction with Fe^3+^. J. Biosci. Bioeng..

[17] Zeng W., Liang Z., Li Z., Bian Y., Li Z., Tang Z., Chen G. (2016). Regulation of poly-γ-glutamic acid production in *Bacillus subtilis* GXA-28 by potassium. J. Taiwan Inst. Chem. Eng..

[18] Feng J., Shi Q., Zhou G., Wang L., Chen A., Xie X., Huang X., Hu W. (2017). Improved production of poly-γ-glutamic acid with low molecular weight under high ferric ion concentration stress in *Bacillus licheniformis* ATCC 9945a. Process Biochem..

[19] Kimura K., Fujimoto Z. (2010). Enzymatic degradation of poly-gamma-glutamic acid. Amino-Acid Homopolymers Occur. Nat..

[20] Suzuki T., Tahara Y. (2003). Characterization of the *Bacillus subtilis ywtD* gene, whose product is involved in γ-polyglutamic acid degradation. J. Bacteriol..

[21] Tian G., Fu J., Wei X., Ji Z., Ma X., Qi G., Chen S. (2014). Enhanced expression of *pgdS* gene for high production of poly–γ–glutamic aicd with lower molecular weight in *Bacillus licheniformis* WX–02. J. Chem. Technol. Biotechnol..

[22] Wang D., Wang H., Zhan Y., Xu Y., Deng J., Chen J., Cai D., Wang Q., Sheng F., Chen S. (2020). Engineering expression cassette of *pgdS* for efficient production of poly-γ-glutamic acids with specific molecular weights in *Bacillus licheniformis*. Front. Bioeng. Biotechnol..

[23] Ojima Y., Kobayashi J., Doi T., Azuma M. (2019). Knockout of *pgdS* and *ggt* gene changes poly-γ-glutamic acid production in *Bacillus licheniformis* RK14-46. J. Biotechnol..

[24] Simon J., van Spanning R.J., Richardson D.J. (2008). The organisation of proton motive and non-proton motive redox loops in prokaryotic respiratory systems. Biochim. Biophys. Acta-Bioenerg..

[25] Li X., Gou X., Long D., Ji Z., Hu L., Xu D., Liu J., Chen S. (2014). Physiological and metabolic analysis of nitrate reduction on poly-gamma-glutamic acid synthesis in *Bacillus licheniformis* WX-02. Arch. Microbiol..

[26] Cai D., Hu S., Chen Y., Liu L., Yang S., Ma X., Chen S. (2018). Enhanced production of poly-γ-glutamic acid by overexpression of the global anaerobic regulator Fnr in *Bacillus licheniformis* WX-02. Appl. Biochem. Biotechnol..

[27] Li X., Yang H., Zhou M., Zhan Y., Liu J., Yan D., Cai D., Chen S. (2021). A novel strategy of feeding nitrate for cost-effective production of poly-γ-glutamic acid from crude glycerol by *Bacillus licheniformis* WX-02. Biochem. Eng. J..

[28] Xu Q., Hou G., Chen J., Wang H., Yuan L., Han D., Hu Q., Jin H. (2021). Heterotrophically ultrahigh-cell-density cultivation of a high protein-yielding unicellular alga *Chlorella* with a novel nitrogen-supply strategy. Front. Bioeng. Biotechnol..

[29] Li X., Yang J., Liu J., Zhang X., Wu W., Yan D., Miao L., Cai D., Ma X., Chen S. (2023). High production of nattokinase via fed-batch fermentation of the γ-PGA-deficient strain of *Bacillus licheniformis*. Fermentation.

[30] Cataldo D., Maroon M., Schrader L.E., Youngs V.L. (1975). Rapid colorimetric determination of nitrate in plant tissue by nitration of salicylic acid. Commun. Soil Sci. Plant Anal..

[31] Nicholas D.J.D., Nason A. (1957). Determination of nitrate and nitrite. Methods Enzymol..

[32] Kongklom N., Shi Z., Chisti Y., Sirisansaneeyakul S. (2017). Enhanced production of poly-γ-glutamic acid by *Bacillus licheniformis* TISTR 1010 with environmental controls. Appl. Biochem. Biotechnol..

[33] Peng Y., Zhang T., Mu W., Miao M., Jiang B. (2016). Intracellular synthesis of glutamic acid in *Bacillus methylotrophicus* SK19. 001, a glutamate–independent poly (γ–glutamic acid)–producing strain. J. Sci. Food Agric..

[34] Cui J., Niu X., Zhang D., Ma J., Zhu X., Zheng X., Lin Z., Fu M. (2023). The novel chitosan-amphoteric starch dual flocculants for enhanced removal of Microcystis aeruginosa and algal organic matter. Carbohydr. Polym..

[35] Härtig E., Jahn D. (2012). Regulation of the anaerobic metabolism in *Bacillus subtilis*. Adv. Microb. Physiol..

[36] Bueno E., Mesa S., Bedmar E.J., Richardson D.J., Delgado M.J. (2012). Bacterial adaptation of respiration from oxic to microoxic and anoxic conditions: Redox control. Antioxid. Redox Signal..

[37] Jiang D., Tikhomirova A., Bent S.J., Kidd S.P. (2016). A discrete role for FNR in the transcriptional response to moderate changes in oxygen by *Haemophilus influenzae* Rd KW20. Res. Microbiol..

[38] Wessel A.K., Arshad T.A., Fitzpatrick M., Connell J.L., Bonnecaze R.T., Shear J.B., Whiteley M. (2014). Oxygen limitation within a bacterial aggregate. MBio.

[39] Wang S., Cao X., Shen M., Guo R., Bányai I., Shi X. (2012). Fabrication and morphology control of electrospun poly (γ-glutamic acid) nanofibers for biomedical applications. Colloids Surf. B Biointerfaces.

[40] Yao J., Jing J., Xu H., Liang J., Wu Q., Feng X., Ouyang P. (2009). Investigation on enzymatic degradation of γ-polyglutamic acid from *Bacillus subtilis* NX-2. J. Mol. Catal. B Enzym..

[41] Zeng J., Jin Y., Liu Z. (2018). Solution scattering study of the *Bacillus subtilis* PgdS enzyme involved in poly-γ-glutamic acids degradation. PLoS ONE.

[42] Feng J., Gao W., Gu Y., Zhang W., Cao M., Song C., Zhang P., Sun M., Yang C., Wang S. (2014). Functions of poly-gamma-glutamic acid (γ-PGA) degradation genes in γ-PGA synthesis and cell morphology maintenance. Appl. Microbiol. Biotechnol..

[43] Abe K., Ito Y., Ohmachi T., Asada Y. (1997). Purification and properties of two isozymes of γ-glutamyltranspeptidase from *Bacillus subtilis* TAM-4. Biosci. Biotechnol. Biochem..

[44] Sha Y., Zhang Y., Qiu Y., Xu Z., Li S., Feng X., Wang M., Xu H. (2018). Efficient biosynthesis of low-molecular-weight poly-γ-glutamic acid by stable overexpression of PgdS hydrolase in *Bacillus amyloliquefaciens* NB. J. Agric. Food Chem..

[45] Sha Y., Huang Y., Zhu Y., Sun T., Luo Z., Qiu Y., Zhan Y., Lei P., Li S., Xu H. (2020). Efficient biosynthesis of low-molecular-weight poly-γ-glutamic acid based on stereochemistry regulation in *Bacillus amyloliquefaciens*. ACS Synth. Biol..

[46] Scoffone V., Dondi D., Biino G., Borghese G., Pasini D., Galizzi A., Calvio C.J. (2013). Knockout of *pgdS* and *ggt* genes improves γ–PGA yield in *B. subtilis*. Biotechnol. Bioeng..

[47] Candela T., Fouet A. (2005). *Bacillus anthracis* CapD, belonging to the γ--glutamyltranspeptidase family, is required for the covalent anchoring of capsule to peptidoglycan. Mol. Microbiol..

[48] Richter S., Anderson V.J., Garufi G., Lu L., Budzik J.M., Joachimiak A., He C., Schneewind O., Missiakas D. (2009). Capsule anchoring in *Bacillus anthracis* occurs by a transpeptidation reaction that is inhibited by capsidin. Mol. Microbiol..

[49] Moud A.A. (2022). Polymer based flocculants: Review of water purification applications. J. Water Process Eng..

[50] Yanagibashi T., Kobayashi M., Omori K. (2019). Application of poly-γ-glutamic acid Flocculant to flocculation–sedimentation treatment of ultrafine cement suspension. Water.

[51] Campos V., Fernandes A.R., Medeiros T.A., Andrade E.L. (2016). Physicochemical characterization and evaluation of PGA bioflocculant in coagulation-flocculation and sedimentation processes. J. Environ. Chem. Eng..

[52] Zheng H., Gao Z., Yin J., Tang X., Ji X., Huang H. (2012). Harvesting of microalgae by flocculation with poly (γ-glutamic acid). Bioresour. Technol..

