# Supplementary material for: Synthesis of Super-High-Viscosity Poly-γ-Glutamic Acid by pgdS-Deficient Strain of Bacillus licheniformis and Its Application in Microalgae Harvesting
Source: Microorganisms. 2024 Nov 22;12(12):2398. doi: 10.3390/microorganisms12122398 (PMC11679365; doi:10.3390/microorganisms12122398)
Supplement: Supplementary file 1 [file microorganisms-12-02398-s001.zip › microorganisms-3273791-supplementary.pdf]

Table S1. Primers used for PCR and qPCR in this study.

Primer name	Sequence of primer (5' to 3')[a]
**Primers for PCR**	
pgdS-AF(*Xba*I)	GC**TCTAGA**AAAGGAACAAACCTCGACTGGA
pgdS-AR	CGAACGATCTGTCAATGTTTTGCGGTTCCCATCCCGAATTG
pgdS-BF	CAATTCGGGATGGAACCGCAAAACATTGACAGATCGTTCG
pgdS-BR(*Sac*I)	C**GAGCTC**CGGGGGAACGGTCCACGA
pgdS-YF	TTTCTACAGCCTCGGCAACT
pgdS-YR	CGGCAAACTGCTCTTACA
T2-F	ATGTGATAACTCGGCGTA
T2-R	GCAAGCAGCAGATTACGC
**Primers for qPCR**	
16s rRNA-F	ACCTAACCAGAAAGCCACGG
16s rRNA-R	GTTTACGGCGTGGACTACCA
ndh-F	TGTCGTCGGTCTTGGTTCTG
ndh-R	ATGAAGTCTGGCTGCTGACC
cydB-F	AGTCGTATTCGCCGTTTTGG
cydB-R	GCTGCCGAAAAATACGACCC
qoxB-F	CCGCCGCACTATAACTTTGC
qoxB-R	CACCGATCAGACCGACGATT
fnr-F	TGACGTCCGATGGCAAAGAA
fnr-R	AAACTCAAACGTCAGCGCAC
narG-F	ATTGCCAAATACGGCCCTGA
narG-R	TAGCGGGCTTCGGCTAAAAA
nasB-F	ACGAATGCCGAAGAGCTGAA
nasB-R	GAAGCGTACACGCAATCGTC
nasD-F	GAGCGACGATGAGATCGTGT
nasD-R	TCAAATTCTGAGCCGAGCGT

[a] The underlines indicate an overlap region for splicing overlap extension PCR (SOE- PCR); Generated restriction site in bold.

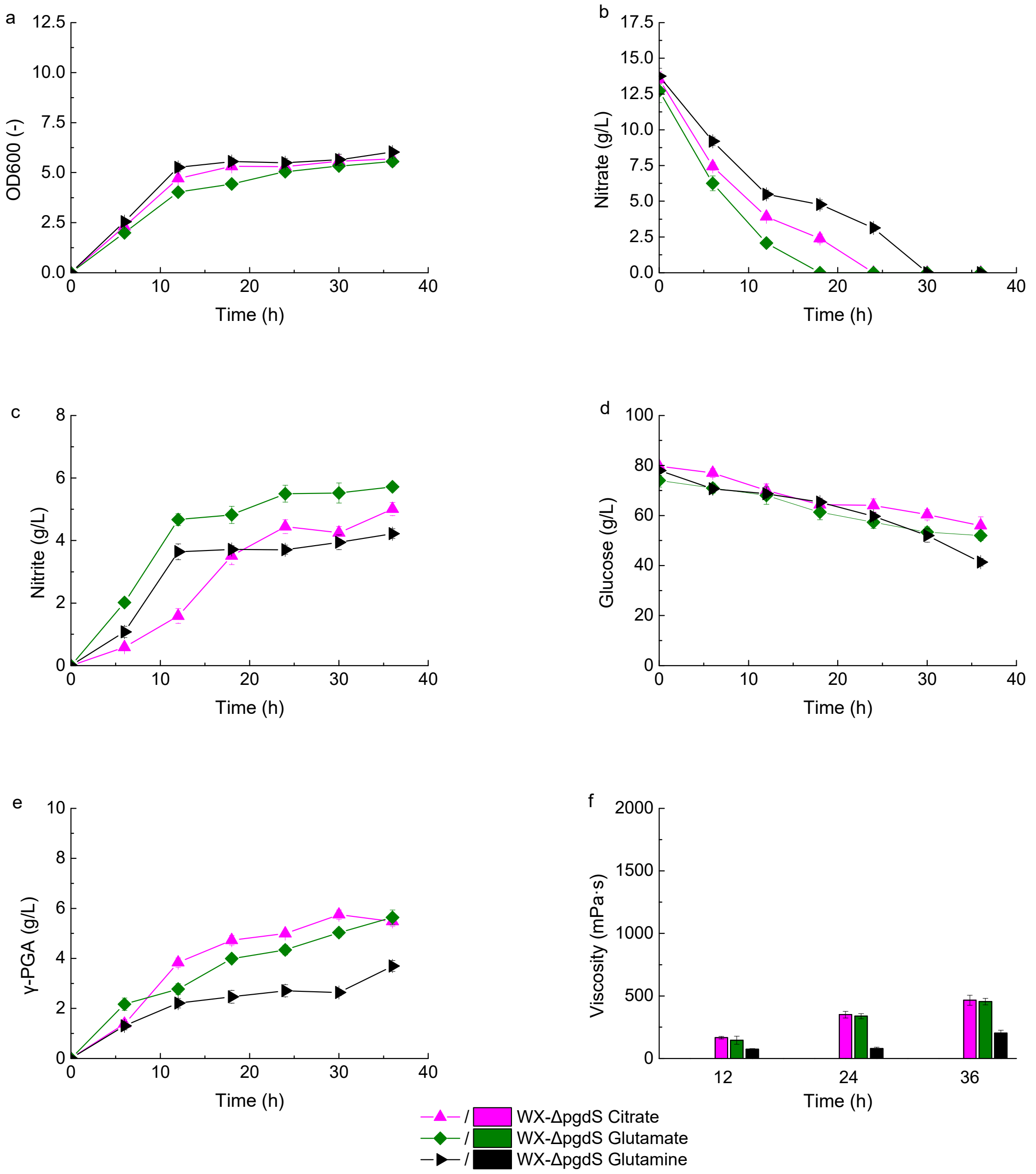

Figure S1. The fermentation profiles of WX-ΔpgdS with using different precursor substrates under the regular oxygen supply conditions. (a) OD600; (b) Nitrate; (c) Nitrite; (d) Glucose; (e) γ-PGA yield; (f) Broth viscosity.

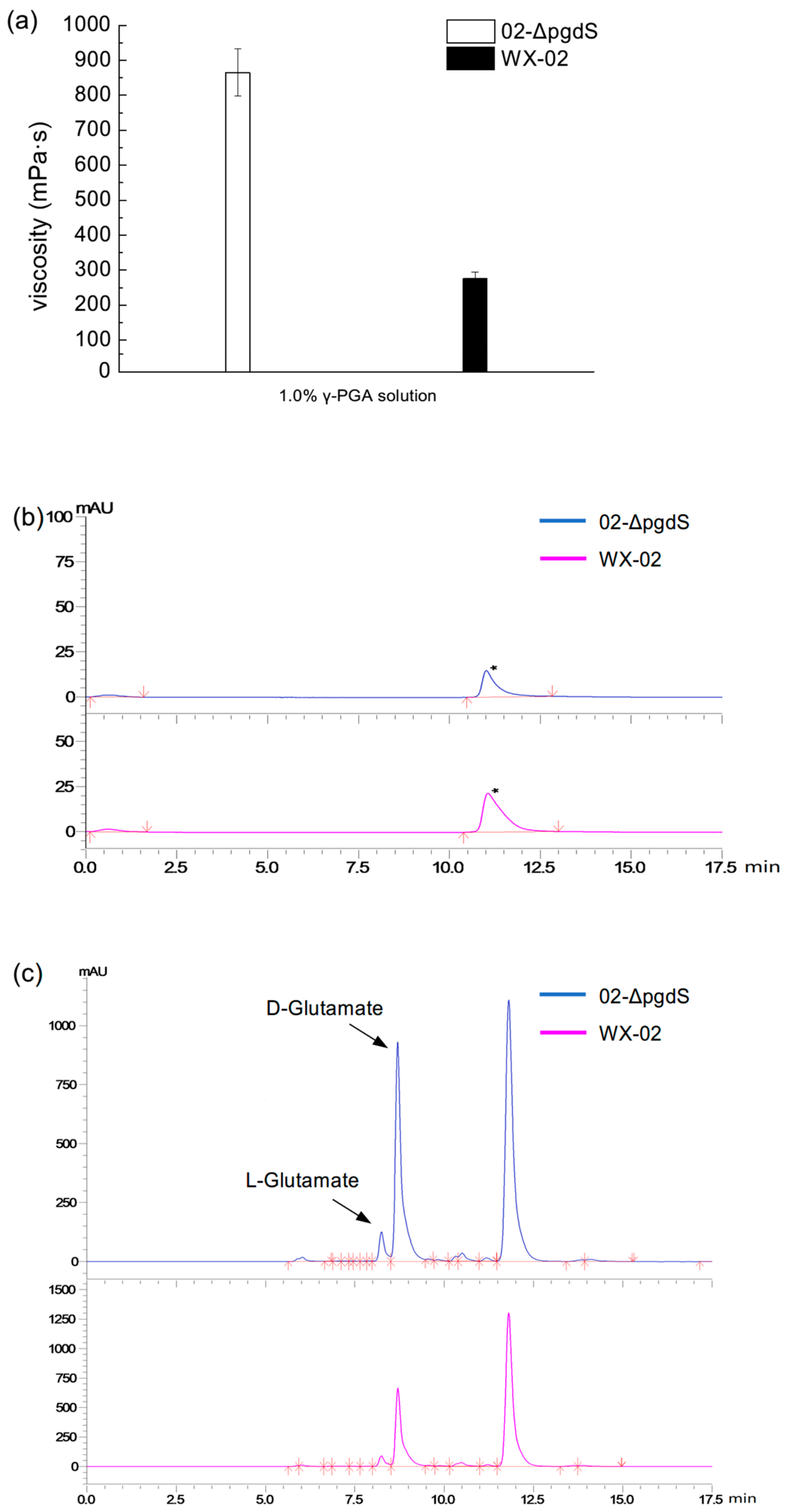

Figure S2. Characteristics assay of γ-PGA produced by WX-ΔpgdS and WX-02. (a) viscosity of 1.0% γ-PGA solution; (b) GPC profile of γ-PGA; (c) The concentration of D/L-glutamate in hydrolyzed γ-PGA.

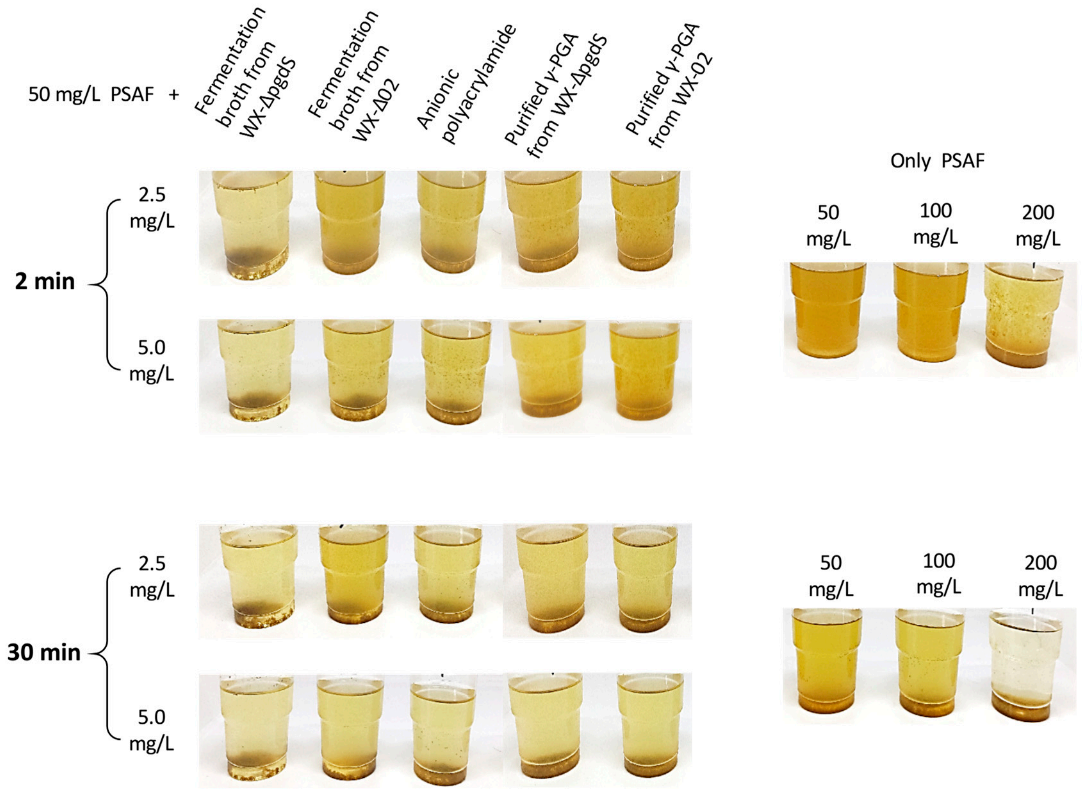

Figure S3. Flocculation of microalgae suspension.
